# Leaden Water-Pipes

**Published:** 1861-01

**Authors:** 


**LEADEN WATER-PIPES.**—It is a well-established fact that persons are constantly losing health and life by the poison introduced into their systems through the water brought to their dwellings by leaden pipes. The first and best precaution is to avoid using the first water drawn, either for drinking or cooking purposes. The longer the faucet has been unturned, the longer should the water be allowed to run to waste—say a minute after a night's disuse. It has recently been suggested by an influential daily paper, that to prevent a useless waste of water in New-York, water-meters should be attached to each building, as in the case of gas. This is a most mischievous suggestion, for then the desire to save expense would cause multitudes to run the risk of lead-poison. The supply of water should be as unrestricted as possible, especially in great cities and crowded dwellings.

To poison the water, the lead must be decomposed; this is done by whatever detains the water in the pipe; not that the water itself decomposes the lead, but wherever water is detained, there is a sediment, which sediment always contains decomposable particles, such as bits of leaves, wood, animal, or other organic matter; even inorganic substances undergo chemical decomposition, and acting on the lead cause corrosion or oxygenation. There are three great efficient precautions which ought to be taken in the introduction of water into dwellings through leaden pipes. First, no particle of mortar or stone or other obstruction should be left in the pipe at the time of its being laid. Second, every possible care should be taken to avoid any indentation of the pipe, especially on its under portion. Third, never allow a short turn in the pipe, no approach to a right angle, but make a segment of as large a circle as possible. The nearer a straight line the better and safer by far. In fact a direct conduit is the only perfect safety, as long as lead is used.

